# Toxic tasting: how capuchin monkeys avoid grasshoppers’ chemical defenses

**DOI:** 10.1007/s10329-024-01133-9

**Published:** 2024-05-25

**Authors:** Henrique P. Rufo, Luiza G. Ferreira, Eduardo B. Ottoni, Tiago Falótico

**Affiliations:** 1 Institute of Psychology, University of São Paulo, São Paulo, Brazil; 2 Capuchin Culture Project, Neotropical Primates Research Group, São Paulo, Brazil; 3 School of Arts, Sciences and Humanities, University of São Paulo, São Paulo, Brazil; 4 Technological Primates Research Group, Max Planck Institute for Evolutionary Anthropology, Leipzig, Germany

**Keywords:** Predation, Chemical defense, Food manipulation, Ontogeny, Food processing

## Abstract

Platyrrhines consume many species of arthropods in the order Orthoptera. Some species of orthopterans can produce chemical defenses that render them toxic or unpalatable and thus act as predator deterrents. These species include the stick grasshoppers (family Proscopiidae), which are widely distributed in the Caatinga biome in northeastern Brazil, which comprises part of the distribution of capuchin monkeys. Capuchin monkeys are omnivores and consume a wide variety of foods, including unpleasant-tasting, potentially toxic items, which they need to learn how to process. We describe the processing of stick grasshoppers (*Stiphra* sp.) by wild capuchin monkeys (*Sapajus libidinosus*) that live in Serra da Capivara National Park, Brazil, and compare how individuals of different age classes handle these potentially toxic food items. *S. libidinosus* predominantly avoided consuming the digestive tract, which contains toxic compounds, when feeding on stick grasshoppers. Immatures took longer than adults to process the stick grasshoppers, indicating that capuchins need to learn how to process the toxic digestive tract of these prey to avoid consuming it.

**Supplementary Information:**

The online version contains supplementary material available at 10.1007/s10329-024-01133-9.

## Introduction

Predator–prey interactions play a crucial role in evolution, with selective pressures being driven by arms race dynamics that act on both morphological and behavioral traits and lead to coevolution (Abrams 2000; Netz et al. 2022; Ridley 2009). Many species of arthropods produce chemical defenses that can render them toxic or unpalatable to certain predators and thus deter the latter from feeding on them (Blum 1981). However, some predators avoid ingesting the toxic parts of an arthropod or are even able to remove them before consuming their prey (Brower and Glazier 1975; Brown and Vasconcelos-Neto 1976; Whitman et al. 1985).

Primates deal with toxic food in a variety of ways. Although they usually avoid eating parts that are toxic, they may actually seek out toxic component(s) for self-medication, as seen in anointing behavior (Alfaro et al. 2012). When they taste food that is bitter, primates may exhibit tongue protrusion and gaping (Steiner et al. 2001). Head-shaking is another behavior exhibited when primates taste bitter substances, which is probably used as a means of quickly expelling the unpleasant-tasting item from the mouth (Steiner et al. 2001) and indicates that there may be a deep evolutionary root for the identification and avoidance of bitter tastes, which are usually indicators of toxicity (Fischer et al. 2005). Herbivorous primates such as mangabeys (*Lophocebus albigena*) usually try to avoid consuming highly toxic food (e.g. tannin-rich plants), and increase their consumption of fruits during periods when the concentrations of tannins decrease (Masette et al. 2015).

However, primates may also actively seek out toxic plants. There is evidence that chimpanzees (*Pan troglodytes*), bonobos (*Pan paniscus*), and gorillas (*Gorilla gorilla*) may self-medicate or gain a prophylactic benefit against gastrointestinal nematodes by consuming plants of no known nutritional value to them that contain secondary compounds that may even be toxic (Huffman 2003; Hart 2005). This shows that primates can identify toxicity in their food and show flexibility in their responses to it.

Capuchin monkeys (genera *Cebus* and *Sapajus*) are omnivores (Fragaszy et al. 2004) and consume a great variety of foods, including potentially toxic ones. However, not every capuchin population deals with the challenges posed by a toxic foodstuff in the same way. For example, cashew nuts (*Anacardium* sp.) contain a toxic and caustic liquid that protects the seed from predation (Sirianni and Visalberghi 2013). Populations of bearded capuchins (*Sapajus libidinosus*) have developed different ways of dealing with this food, with some avoiding eating cashew nuts altogether (Falótico et al. 2018), and others using a rubbing technique (Sirianni and Visalberghi 2013) or stone tools (Luncz et al. 2016) to remove the toxic liquid before consuming the seed. Capuchins also make use of toxic substances for self-medication.

Orthopterans are a good source of protein for insectivorous and omnivorous primates (Raubenheimer and Rothman 2013). Female insects can store great amounts of fat during the reproductive period (Arrese and Soulages 2010). As a consequence of their high fat contents, Orthoptera, and mostly grasshoppers, are consumed by a variety of platyrrhines, such as members of the genera *Aotus*, *Cebuella*, *Cebus*,* Cacajao*,* Callithrix*, *Lagothrix*, *Leontocebus*, *Saimiri*, and *Sapajus* (Izawa 1978a, b, 1979; Janson and Boinski 1992; Nickle and Heymann 1996; Lima and Ferrari 2003; Heymann and Buchanan-Smith 2007; Melin et al. 2007; Madden et al. 2010; Jesus et al. 2022). However, some grasshoppers are toxic as part of their predation-deterrence strategy (Blum 1981). The stick grasshoppers (family Proscopiidae) are among these toxic insects. These insects are not toxic per se, but when they consume certain toxic plants, they retain some of the toxic compounds in their digestive tract, and may become unpalatable and toxic as a consequence (Bidau 2014).

Stick grasshoppers are widespread in the capuchin monkeys’ distribution (Lecoq and Magalhães 2006). Members of the stick grasshopper genus *Stiphra*, which are well adapted to semi-arid environments, were first identified in the Caatinga biome in the northeastern regions of Brazil in the late 1930s (Mello-Leitão 1939). Although a potential food for capuchin monkeys, the monkeys may need to adequately process these grasshoppers to avoid ingesting unpalatable (and potentially toxic) parts. As the toxic contents of these insects are located in their digestive tract, we expect capuchins that are aware of this to avoid consuming the parts of their prey that contain it.

In this report, we aim to describe the processing of stick grasshoppers (*Stiphra* sp.) by wild capuchin monkeys (*Sapajus libidinosus*) that live in Serra da Capivara National Park (SCNP), northeastern Brazil, and compare how individuals of different age classes handle these food items. Younger individuals were expected to be less proficient in removing the undesirable digestive tract and thus to consume it more frequently, while older individuals were expected to be more successful in removing and thus avoiding these unpalatable parts of their prey.

## Methods

The Pedra Furada group of bearded capuchin monkeys (*S. libidinosus*) has been studied since 2007 (Falótico and Ottoni 2016, 2023). These animals live in SCNP (8.8333°S, 42.5500°W) in the Caatinga biome (semi-arid savannah) in northeastern Brazil. The group comprised 40 individuals at the time of this study.

**Fig. 1:**
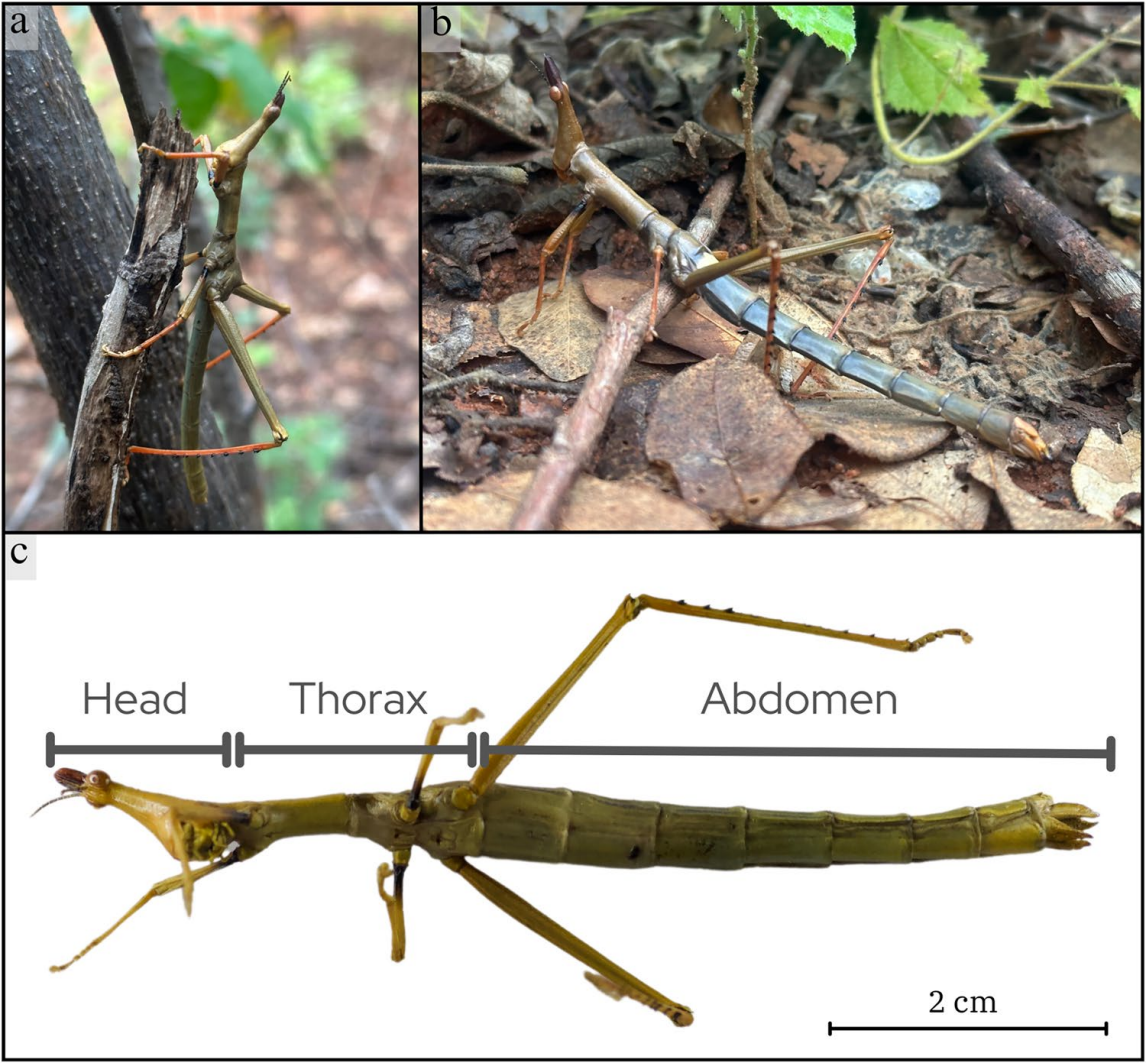
**a**, **b** Stick grasshoppers (*Stiphra* sp.) in their natural habitat. **c** Head, thorax, and abdomen of a stick grasshopper

We observed and video-recorded events of capuchin monkeys capturing, processing, and eating giant jumping stick grasshoppers (*Stiphra* sp., Orthoptera, Proscopiidae; Fig. 1) from 23 March to 18 April 2023, during the grasshopper mating season. The observations were done ad libitum in the course of behavioral sampling of the group. We followed the capuchin group from dawn to dusk.

We video-recorded events of capuchin monkeys manipulating and consuming individual grasshoppers (Online Resource 1). For each event, we noted the following behaviors: grab prey; hold prey (duration in seconds); bite prey (with the added detail of which part of the insect’s body was bitten); consume prey (with the added detail of the body part consumed); spit out (with the added detail of the body part spat out); drop prey; digestive tract extraction (defined as part of the digestive tract from the thorax or abdomen, or both, becoming visible, with the added detail of it being either eaten or dropped), lick intestines (with the added detail of licking off the fat or mucosa). We coded the behaviors by using the software Boris, version 8.21 (Friard and Gamba 2016). We also noted capuchin sex, age, and individual identification for most of the adult monkeys (the immatures were not identifiable), and classified the individuals into immature (< 3 years old) or adult/mature (> 3 years old) age classes.

To compare the processing of their prey by adult and immature capuchins, we calculated (1) total manipulation time (from first bite to when all of the prey had been consumed or dropped); (2) the duration of extraction of digestive tract (from the beginning of its extraction until the moment at which it was dropped); (3) frequency with which a particular part of the prey was first bitten into; (4) frequency with which digestive tract was removed from a specific part of the prey; (5) frequency of occurrence of fat licking; (6) frequency with which different parts of the prey were eaten: (7) frequency with which a particular part of the prey was dropped during each event.

To compare the duration of manipulation of their grasshopper prey by adults and immatures, we fitted a general linear model (GLM) model, with time of manipulation as the dependent variable and age class as the independent one. We used a gamma distribution model. The analyses were performed in R (R Core Team 2023) using the lme4 package (Bates et al. 2015). The raw data and the R script are given in Online Resources 2 and 3.

## Results

We observed a total of 92 events of 17 capuchins manipulating and eating stick grasshoppers. Of these individuals, six were adult males, five were adult females, and six were immatures. We were able to observe the manipulation in detail for 50 events, 44 of which were recorded for adults and six for immatures (Table 1; Online Resource 1). For all events, the prey was at least partly consumed. The insect digestive tract was removed and discarded in 94% of the events (Table 1), and the abdomen of the insect removed and discarded in the remaining 6% of events (*n* = 3). Adults and immatures manipulated their prey for a similar amount of time (GLM, *t* = − 0.552, *p* = 0.58; Table 1). However, immatures took longer than adults to commence removing the insect’s digestive tract after the first bite (GLM, *t* = − 2.207, *p* = 0.03; Table 1).

**Table 1:** Number of events (percentage in parentheses) and average time of manipulation from the first bite taken from the prey until its consumption, with or without digestive tract extraction, including time until the former commenced

Age	Manipulation with digestive tract extraction (s)			Manipulation without digestive tract extraction (s)	
	TMT^1^ (mean ± SD)	TSDTE^2^ (mean ± SD)	*n* (%)	TMT (mean ± SD)	*n* (%)
Adults	14.5 ± 10.7	3.0 ± 2.9	41 (82%)	9.5 ± 8.0	3 (6%)
Immatures	17.0 ± 8.4	6.3 ± 4.9	6 (12%)	–	–
Total	14.8 ± 10.4	3.4 ± 3.3	47 (94%)	9.5 ± 8.0	3 (6%)

*TMT* Total manipulation time,* TSDTE* time until start of digestive tract extraction

**Fig. 2a–f:**
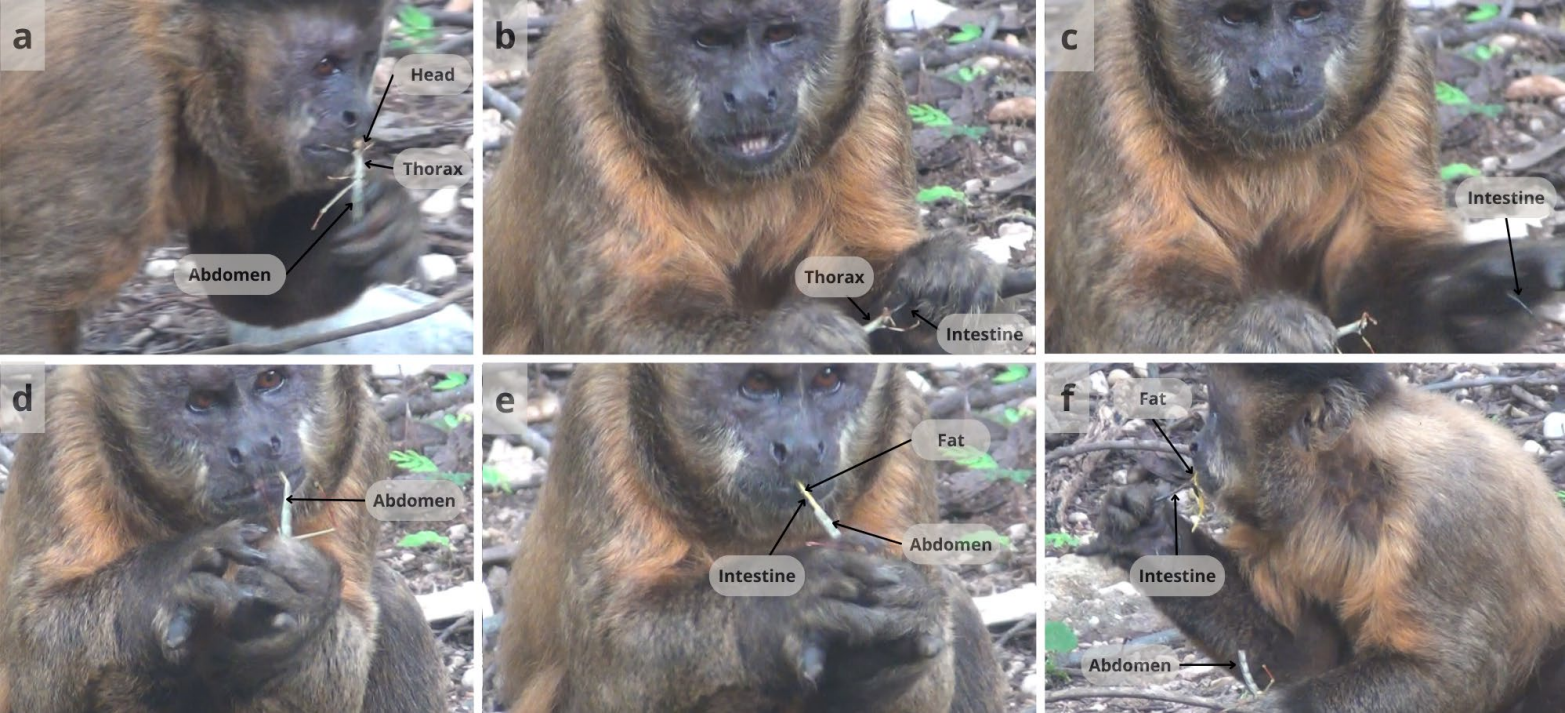
An example of the typical sequence in which grasshoppers were processed by the capuchin monkeys (here an adult male), showing extraction of digestive tract from both the thorax and abdomen. Processing started with the first bite to the head (**a**), followed by extraction of digestive tract from the thorax (**b**), and then discarding a portion of the digestive tract (**c**). The next bites were to the abdomen (**d**), followed by extraction of the intestines through the wound (**e**), and licking the fat from the intestines (**f**)

In most events involving adults, the first bite was to the head (Fig. 2a), followed by the abdomen and thorax/legs (Table 2). In contrast, immature individuals took their first bite more often from the thorax/legs, followed by the head (Table 2). There were no events where immatures took their first bite from the abdomen (Table 2). Both immatures and adults removed digestive tract more frequently from the abdomen than from the the head/thorax. Only adults removed digestive tract from both the thorax (Fig. 2b, c) and abdomen (Fig. 2d, e; Table 2).

**Table 2:** Number (percentage in parentheses) of events where the head, thorax/legs or abdomen received the first bite, and where digestive tract was extracted from the thorax or abdomen or both

Age class	First bite			Digestive tract extraction		
	Head	Thorax/legs	Abdomen	Thorax	Abdomen	Thorax and abdomen
Adults	32 (74%)	5 (12%)	6 (14%)	13 (32%)	23 (56%)	5 (12%)
Immatures	2 (40%)	3 (60%)	0 (0%)	1 (17%)	5 (83%)	0 (0%)

The process of removing and then discarding the digestive tract was shorter in adults than in immatures (Table 3). Adults took, on average, longer to extract digestive tract from the abdomen than from the thorax (Table 3).

**Table 3:** Average time taken by adults and immatures to complete the process of removing and then discarding digestive tract from the thorax or abdomen or both

Age	Thorax		Abdomen		Thorax and abdomen		Total	
	Average time (s)	*n*	Average time (s)	*n*	Average time (s)	*n*	Average time (s)	*n*
Adults	0.7 ± 0.3	13	5.9 ± 7.5	23	5.5	5	4.2 ± 6.1	41
Immatures	0.7	1	12.9 ± 16.3	5	–	0	10.9 ± 15.4	6

When the intestines were removed from the abdomen, the associated fat was also removed (Fig. 2e). Sometimes the monkeys licked this fat (Fig. 2f), which increased the time it took them to discard the intestines. When there was no fat licking, adults took more time than immatures to discard the intestines (Table 4). In two of these events, the adults ate other parts of the insect after they had started to extract the intestines but before they discarded them, and took longer to process the insects before consuming them (Table 5). However, when the immature monkeys licked the associated fat before discarding the intestines, they took longer than the adults to complete this process.

**Table 4:** Number of events and average time taken by adults and immatures to extract the intestines from the abdomen and discard them with or without licking of associated fat

Age	Fat licking (s)	*n*	No fat licking (s)	*n*
Adults	8.7 ± 9.0	12	2.8 ± 3.8	11
Immatures	21.1 ± 16.7	3	0.6 ± 0.02	2

**Table 5:** Number of events and average time taken by adults and immatures to extract the intestines from the abdomen and discard them with and without consumption of other parts of the insect during the same period

Age	Other parts consumed during intestine extraction		Other parts not consumed during intestine extraction		Total	
	Time (s)	*n*	Time (s)	*n*	Time (s)	*n*
Adult	8.3 ± 8.1	2	1.6 ± 0.9	9	2.8 ± 3.8	11
Immature	–	0	0.6 ± 0.02	2	0.6 ± 0.02	2

In none of the events analyzed was an individual observed showing any disgust behavior toward its grasshopper prey, although there was one event (observed by HPR) in which an immature individual displayed a distasteful expression when his lips made contact with the grasshopper’s intestines.

## Discussion

*S. libidinosus* notably avoided consuming the digestive tract when feeding on the stick grasshoppers, which presumably contains compounds that act as chemical defenses. Similarly, when feeding on cashew nuts, capuchins use strategies to avoid consuming the caustic liquid within the shell (Sirianni and Visalberghi 2013; Luncz et al. 2016).

Squirrel monkeys (genus *Saimiri*) also avoided eating the digestive tract of orthopterans by removing it from these prey (Janson and Boinski 1992). However, *Sapajus apella* and species of *Cebus* (*Cebus albifrons* and *Cebus olivaceus*) were reported to be less meticulous than squirrel monkeys in removing the digestive tract of orthopterans (Janson and Boinski 1992). Izawa (1978a) also noted that tamarins (*Saguinus nigricollis*) consumed all of the entrails of grasshoppers, even after removing them from the insects; however, the grasshoppers were not identified and may not have contained toxic compounds. The *S. libidinosus* that were observed (TF and HPR) at SCNP feeding on non-toxic grasshoppers (*Tropidacris* spp.) ate the entire insect.

Saddle-back tamarins (*Saguinus fuscicollis*), moustached tamarins (*Saguinus mystax*), and black-lion tamarins (*Leontopithecus chrysopygus*) also feed on Proscopiidae (Carvalho et al. 1989; Nickle and Heymann 1996); however, how the tamarins processed Proscopiidae was not described in detail in these reports. Carvalho et al. (1989) reported that black-lion tamarins avoid ingesting too much chitin by removing the legs of grasshoppers, or regurgitating them if they are ingested. Our data indicate that the adult capuchins rarely bit the legs of the grasshoppers first, which may indicate that they also avoided ingesting too much chitin.

The adult capuchins preferred to bite the head of the grasshopper first, while the immatures did not show a preference for the head or thorax for their first bite. Tamarins always bite the head first when feeding on grasshoppers and other orthopterans (Izawa 1978a; Nickle and Heymann 1996). Directing their first bite to the head may be a way of tamarins killing their prey quickly, or a means of avoiding being bitten by arthropods in general (Nickle and Heymann 1996).

The observed pattern of grasshopper digestive tract removal does not appear to be group specific in *S. libidinosus*. For example, in another group of *S. libidinosus* at SCNP, an adult male was observed (HPR) capturing a grasshopper, removing its intestines, and then consuming it. Another population of *S. libidinosus* living at Ubajara National Park, Brazil, that preys on *Stiphra* sp. were observed (TF and LGF) processing grasshoppers in the same way, i.e. removing the digestive tract from the thorax or abdomen before eating the prey. The process of removing digestive tract from grasshoppers with chemical defenses appears to be similar among the primates that eat them. In addition, adult Central American squirrel monkeys (*Saimiri oerstedii*) also remove the intestines of grasshoppers and caterpillars before consuming them (Boinski and Fragazy 1989).

With respect to differences between adult and immature *Saimiri*, the latter have more difficulty processing toxic caterpillars and grasshoppers (Boinski and Fragaszy 1989), and may be injured as a result of touching the toxic setae of a caterpillar, or have to vomit after inadvertently eating toxic prey. In the present study, immatures took longer than adult capuchins to process stick grasshoppers. At times, the monkeys ate the fat that is associated with the intestines. However, immatures took longer than adults to completely process the intestines, as they had more difficulty separating the fat from them, and spent more time licking the fat off them. Immatures and adults were observed avoiding touching the intestines with their lips, with one exception, where the immature made a distasteful face and spat the intestines out.

In summary, the differences observed here between immature and adult capuchins in processing stick grasshoppers indicate that they need to learn how to process and extract as much food as possible from these prey. Foraging of capuchins for Proscopiidae mainly occurs during the grasshopper mating season, which offers only a small window in which immature capuchins can learn how to process these prey. This process may involve asocial and/or social learning. Social learning is important in capuchin monkey societies (Ottoni and Izar 2008), as it plays a major role in the acquisition of tool-use skills (Ottoni et al. 2005; Resende et al. 2008; Falótico et al. 2021) and in maintaining behavioral traditions (Ottoni 2015). The existence (or absence) and ontogeny of social learning in the development of skills for the manipulation of stick grasshoppers, and food items in general that require processing, need to be further examined to gain a better understanding of how monkeys deal with these types of food throughout their lives.

### Supplementary Information

Below is the link to the electronic supplementary material.

Supplementary file1 (MP4 63180 KB)

Supplementary file2 (CSV 6 KB)

Supplementary file3 (TXT 1 KB)

## Data Availability

The data supporting this article are available as supplementary material.
